# A helix tacking system for the management of colonic anastomotic dehiscence, after a laparoscopic left colectomy

**DOI:** 10.1055/a-2760-9005

**Published:** 2026-01-08

**Authors:** Vincent VandenDriessche, Eda Kaya, Patrick Yengue, Maxence Lefebvre

**Affiliations:** 1 82451 Department of Gastroenterology, Centre Hospitalier de Wallonie Picarde, Tournai, Belgium

The endoscopic helix tacking system is increasingly used to treat mucosal defects, particularly following endoscopic submucosal dissection or endoscopic mucosal resection 1 . Here, we aim to demonstrate the feasibility of using this tacking system to manage an anastomotic dehiscence (AD) after oncologic laparoscopic left colectomy. AD is defined as the separation of sutures occurring within 30 days after colectomy 2 . When dehiscence leads to bowel leakage, it can result in pelvic abscesses, peritonitis, or sepsis 3 . The current standard of care includes a diverting colostomy or ileostomy along with the drainage of the surgical site infection. In recent years, however, minimally invasive endoscopic approaches, such as endoluminal vacuum therapy (EVT), have shown effectiveness in managing colonic dehiscence 2 . We present the case of a 69-year-old male patient who underwent laparoscopic left colectomy with an end-to-end colorectal anastomosis, performed 18 cm from the anal margin, to treat colonic adenocarcinoma. Within 2 months, he developed an AD affecting approximately a quarter of the anastomosis (approximately 15 mm), connected to a small cavity measuring 15–20 mm in diameter. The patient initially received EVT, which reduced the orifice size from 15 to 10 mm within 2 weeks. However, complete closure of the fistulising orifice was ultimately achieved with the use of an endoscopic helix tacking system. Closure was accomplished by placing four tacks around the defect in a “Z” pattern ( Fig. 1 and Fig. 2 **a** ), pulling the thread attached to the tacks, and securing the suture with a cinch device to lock the suture and cut the thread ( Fig. 2 **b** ). Six-week follow-up confirmed successful endoscopic closure using X-MAN (heliX tacking system for the Management of colonic ANastomotic dehiscence; Fig. 2 **c** ). This case demonstrates the safe and effective management of post-colectomy anastomotic dehiscence using the X-MAN ( Video 1 ).

**Fig. 1:**
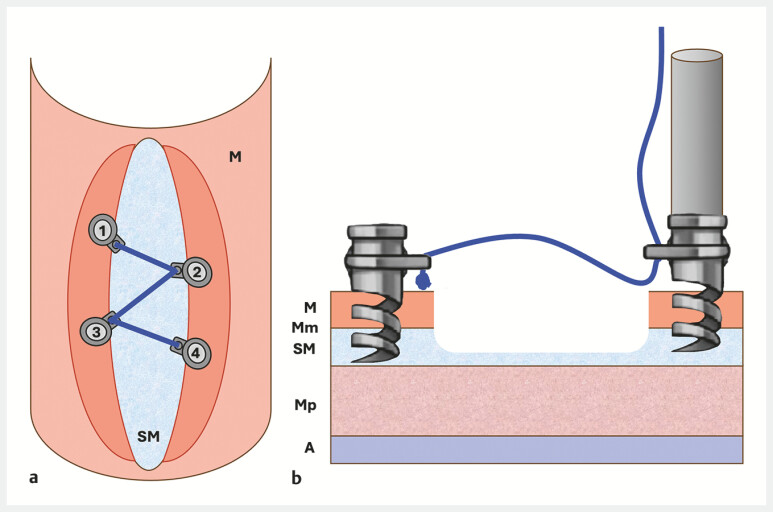
Schematic illustration of the helix tacking system showing a sequential deployment of four helix tacks around a mucosal lesion: **a** Placement of four tacks around the defect in a “Z” pattern. **b** Transmural section of a mucosal defect showing the deployment of helix tacks around a mucosal lesion. A, adventitia; M, mucosa; Mm, muscularis mucosa; Mp, muscularis propria; SM, submucosa.

**Fig. 2:**
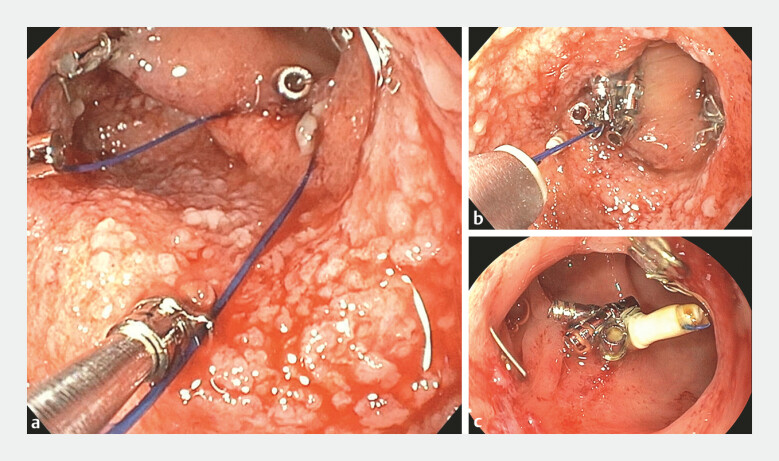
**a** Closure was accomplished by placing four tacks around the defect in a “Z” pattern. **b** Securing the suture with a cinch device to lock the suture and cut the thread. **c** Follow-up at 6 weeks confirmed successful endoscopic closure using the X-MAN (heliX tacking system for the Management of colonic ANastomotic dehiscence).

A helix tacking system for the management of colonic anastomotic dehiscence after a laparoscopic left colectomy.Video 1

Endoscopy_UCTN_Code_TTT_1AQ_2AK

## References

[1] Mohapatra S Fukami N Follow-up outcomes of mucosal defect closures after endoscopic resection using a helix tacking system and endoclips VideoGIE 2022 7 268 272 10.1016/j.vgie.2022.03.002 35815167 PMC9263876

[2] Cwaliński J Hermann J Paszkowski J Dehiscence of colorectal anastomosis treated with non invasive procedures Wideochir Inne Tech Maloinwazyjne 2023 18 128 134 37064554 10.5114/wiitm.2022.121701 PMC10091912

[3] Daams F Slieker JC Tedja A Treatment of colorectal anastomotic leakage: results of a questionnaire amongst members of the Dutch Society of Gastrointestinal Surgery Dig Surg 2012 29 516 521 10.1159/000346348 23485790

